# *In silico* Cell Therapy Model Restores Failing Human Myocyte Electrophysiology and Calcium Cycling in Fibrotic Myocardium

**DOI:** 10.3389/fphys.2021.755881

**Published:** 2022-01-03

**Authors:** Katherine G. Phillips, Irene C. Turnbull, Roger J. Hajjar, Kevin D. Costa, Joshua Mayourian

**Affiliations:** ^1^Cardiovascular Research Institute, Icahn School of Medicine at Mount Sinai, New York, NY, United States; ^2^Phospholamban Foundation, Amsterdam, Netherlands; ^3^Department of Pediatrics, Boston Children’s Hospital, Boston, MA, United States; ^4^Department of Pediatrics, Harvard Medical School, Boston, MA, United States; ^5^Department of Pediatrics, Boston University, Boston, MA, United States; ^6^Department of Pediatrics, Boston Medical Center, Boston, MA, United States

**Keywords:** human mesenchymal stem cell (hMSC), cardiac cell therapy, heterocellular coupling, paracrine signaling, heart failure, computational modeling, myocardial fibrosis, cardiomyocyte electrophysiology

## Abstract

Myocardial delivery of human c-kit^+^ cardiac interstitial cells (hCICs) and human mesenchymal stem cells (hMSCs), an emerging approach for treating the failing heart, has been limited by an incomplete understanding of the effects on host myocardium. This computational study aims to model hCIC and hMSC effects on electrophysiology and calcium cycling of healthy and diseased human cardiomyocytes (hCM), and reveals a possible cardiotherapeutic benefit independent of putative regeneration processes. First, we developed an original hCIC mathematical model with an electrical profile comprised of distinct experimentally identified ion currents. Next, we verified the model by confirming it is representative of published experiments on hCIC whole-cell electrophysiology and on hCIC co-cultures with rodent cardiomyocytes. We then used our model to compare electrophysiological effects of hCICs to other non-excitable cells, as well as clinically relevant hCIC-hMSC combination therapies and fused hCIC-hMSC CardioChimeras. Simulation of direct coupling of hCICs to healthy or failing hCMs through gap junctions led to greater increases in calcium cycling with lesser reductions in action potential duration (APD) compared with hMSCs. Combined coupling of hCICs and hMSCs to healthy or diseased hCMs led to intermediate effects on electrophysiology and calcium cycling compared to individually coupled hCICs or hMSCs. Fused hCIC-hMSC CardioChimeras decreased healthy and diseased hCM APD and calcium transient amplitude compared to individual or combined cell treatments. Finally, to provide a theoretical basis for optimizing cell-based therapies, we randomized populations of 2,500 models incorporating variable hMSC and hCIC interventions and simulated their effects on restoring diseased cardiomyocyte electrophysiology and calcium handling. The permutation simulation predicted the ability to correct abnormal properties of heart failure hCMs in fibrotic, but not non-fibrotic, myocardium. This permutation experiment also predicted paracrine signaling to be a necessary and sufficient mechanism for this correction, counteracting the fibrotic effects while also restoring arrhythmia-related metrics such as upstroke velocity and resting membrane potential. Altogether, our *in silico* findings suggest anti-fibrotic effects of paracrine signaling are critical to abrogating pathological cardiomyocyte electrophysiology and calcium cycling in fibrotic heart failure, and support further investigation of delivering an optimized cellular secretome as a potential strategy for improving heart failure therapy.

## Introduction

Heart failure remains a leading cause of morbidity and mortality in Western countries (Benjamin et al., 2017). While current medical management improves symptoms and prolongs life, it does not fully address the underlying pathophysiology. Emerging cell-based therapies—theorized to protect, repair, or even replace diseased myocardium—represent promising treatments that are being actively developed (Sanganalmath and Bolli, 2013; Mathiasen et al., 2019). For example, treating ischemic cardiomyopathy patients by injecting bone marrow-derived human mesenchymal stem cells (hMSCs) induces angiogenesis and decreases myocardial infarction scar size by 30–50% (Hare et al., 2012; Heldman et al., 2014; Karantalis et al., 2014). More recently, Mathiasen et al. (2019) showed that 12 months after transendocardial injection of autologous hMSCs into ischemic heart failure patients, there was a significant improvement in left ventricular ejection fraction and quality of life relative to placebo (Bolli and Kahlon, 2020). Other cells tested include c-kit^+^ cardiac interstitial cells (hCICs); despite recent evidence revealing these cells do not possess regenerative capacity (Li et al., 2018), animal studies have consistently shown that hCIC-treatment after acute myocardial infarction can, like hMSCs, beneficially affect left ventricular remodeling and dysfunction (Tang et al., 2016).

Evidence that hMSCs regulate stem cell niches (Hatzistergos et al., 2010; Mendez-Ferrer et al., 2010) motivated Williams et al. (2013) to deliver combinations of hMSCs and hCICs to pigs with post-ischemic cardiomyopathy, which resulted in a twofold greater reduction in infarction size compared to either cell type delivered alone. This potential synergy has similarly inspired development of CardioChimeras (CCs), a hybrid cell line that fuses molecular and phenotypic properties of CICs and MSCs (Quijada et al., 2015; Firouzi et al., 2020). A related clinical trial (CONCERT-HF) has recently released their phase II results and found that both individual and combined treatment with hCICs and hMSCs lead to improvements in clinical outcomes at 12 months, with the most promising results overall from the combination therapy (Bolli et al., 2021). However, therapeutic efficacy continues to be limited by an incomplete understanding of the mechanisms of action—predominantly involving paracrine signaling (PS) and heterocellular coupling (HC)—by which these cells impact the electrophysiology and calcium cycling of healthy and failing human cardiomyocytes (hCMs) (Menasche, 2018).

For example, in addition to promoting angiogenesis and reducing fibrosis (Ranganath et al., 2012), hMSC PS alters excitation-contraction coupling of cardiomyocytes (Mayourian et al., 2018a), causing increased expression of key calcium cycling genes—including sarcoendoplasmic reticulum calcium-ATPase (SERCA) and L-type calcium channel (LTCC)—and thereby increasing cardiomyocyte calcium transient amplitude and contractility (Desantiago et al., 2012; Hwang et al., 2012; Mayourian et al., 2017). By contrast, when studying the paracrine effects of hCICs, Smit et al. (2017) observed no significant changes in rat cardiomyocyte action potential morphology, and others reported the exosomal component of the hCIC secretome to have limited uptake by cardiomyocytes with no significant effect on cardiomyocyte calcium cycling (Gray et al., 2015; Agarwal et al., 2017).

On the other hand, hMSC HC is known to alter cardiomyocyte action potential duration (APD) and tissue-level conduction velocity (Chang et al., 2006; Mayourian et al., 2016). Similarly, hCIC HC was recently found to decrease rat cardiomyocyte upstroke velocity, depolarize resting membrane potential (RMP), and prolong APD (Smit et al., 2017), and human engineered cardiac tissues supplemented with 10% hCICs displayed significant increases in contractility and markers of cardiomyocyte maturation (Murphy et al., 2019). Clearly, there is a complex and cell type-dependent interactome between cardiomyocytes and these non-excitable cells that is difficult to elucidate experimentally. *In silico* models offer an inexpensive, efficient, and precisely controlled alternative to investigate the mechanisms underlying such cellular interactions.

To this end, this computational study aims to provide a theoretical basis for designing cell-based cardiotherapies by simulating PS and HC effects of hCICs and hMSCs on healthy and failing cardiomyocyte action potential and calcium transient waveforms. First, we develop and validate a novel mathematical model of hCIC electrophysiology based on published experimental data (Zhang et al., 2014). We then apply this model to simulate HC effects of hCICs on cardiomyocytes of multiple species, and using our published hMSC electrophysiology model (Mayourian et al., 2016, 2017) we compare hCIC vs. hMSC and cardiac fibroblast (CF) HC effects on cardiomyocyte action potential and calcium transient waveforms. In addition, we simulate HC effects of clinically relevant combinations of hCICs and hMSCs, as well as hCIC-hMSC fused CardioChimeras. Finally, we generate a population of 2,500 different hMSC and hCIC delivery conditions in fibrotic and non-fibrotic substrates of heart failure hCMs to determine whether functional restoration to healthy hCM properties is theoretically possible, and if so, the crucial delivery parameters that would be required.

## Materials and Methods

All data, code, methods, and study materials are available upon request by contacting the corresponding authors.

### Mathematical Electrophysiology Models of Human c-Kit^+^ Cardiac Interstitial Cells and Other Non-excitable Cells

The differential equation for an uncoupled hCIC transmembrane voltage over time is defined as:


(1) $$\frac{{\text{dV}}_{\text{hCIC}}}{\text{dt}}=-\frac{{\text{I}}_{\mathrm{ion},\mathrm{hCIC}}}{{\text{C}}_{m,\mathrm{hCIC}}}$$

where V_*hCIC*_ is the time-dependent transmembrane voltage [i.e., V_*hCIC*_ = V_*hCIC*_(t)], t is time, C_*m*,*hCIC*_ is the hCIC membrane capacitance (set as 42.5 pF based on experimental data) (Zhang et al., 2014), and I_ion,*hCIC*_ is a function of voltage and time [i.e., I_*ion,hCIC*_ = I_*ion,hCIC*_(V_*hCIC*_, t)] and is the sum of the active ionic currents—namely large-conductance Ca^2+^-activated K^+^ (I_*KCa*_), inward rectifying K^+^ (I_*Kir*_), transient outward K^+^ (I_*to*_), Na^+^ (I_*Na*_) and leakage (I_*leak*_) currents—weighted by their reported prevalence (Zhang et al., 2014).

Note that Equation 1 is part of a system of ordinary differential equations (see Supplementary Table 1 for details) that is explicitly a function of voltage and ion channel activation/inactivation variables and implicitly a function of time. As is typical in the field, a finite difference method is used to numerically integrate over time to determine the implicit time dependence of all variables within the system of ordinary differential equations. All single-cell models were numerically integrated with MATLAB’s stiff ordinary differential equation solver (ode15s) until steady state was achieved, defined as 500 cycles (which achieved less than ± 0.1 mV in consecutive beats for a population of cardiomyocytes coupled to fibroblasts, hCICs, and hMSCs in various combinations). Similar methodology and rationale was used for solving Equations (2) and (3) below. As a primer for such electrophysiology model development, see our recent methods paper (Mayourian et al., 2018c).

**FIGURE 1:**
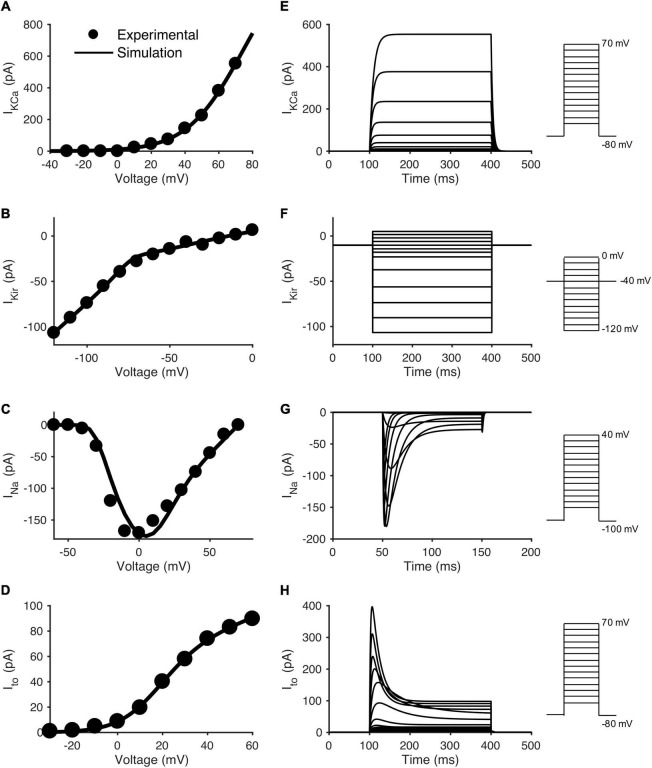
Data fitting and model simulation of hCIC ionic currents. Comparison of experimental and modeled current-voltage (I-V) plots for individual hCIC ion channels, and the resulting voltage-clamp simulations. The left column shows simulated I-V curves for **(A)** large-conductance Ca^2+^-activated K^+^ channel (I_*KCa*_), **(C)** Na^+^ channel (I_*Na*_), and **(D)** transient outward channel (I_*to*_) currents, together with mean experimental data from Zhang et al. (2014). Note that **(B)** Inward rectifying K^+^ channel (I_*Kir*_) was directly fitted to mean I-V experimental data from Zhang et al. (2014), as it does not have activation/inactivation parameters. The right column shows voltage-clamp simulations for the respective channel currents: **(E)** I_*KCa*_, **(F)** I_*Kir*_, **(G)** I_*Na*_, and **(H)** I_*to*_. Note that **(C)** depicts the maximal amplitude I_*Na*_, whereas **(D)** depicts the steady state I_*to*_ current. Voltage step protocols are inset to the right of each voltage-clamp simulation in accordance with the protocols used by Zhang et al. (2014).

To model individual currents in order to develop the hCIC model, published experimental hCIC electrophysiology data from Zhang et al. (2014) was digitized. Unless explicitly provided in their work: (1) steady state activation and inactivation parameters were derived from steady-state current-voltage (I-V) plots; (2) activation kinetic parameters were derived from voltage-clamp data, and were defined as time to reach 63% of the maximum current; and (3) inactivation kinetic parameters were derived from voltage-clamp data, and were defined as time to reach 63% of return from maximal current to steady-state. See Supplementary Figures 1–3 for the digitized data and model fits for each of the voltage-dependent activation/inactivation steady-state and kinetic parameters. Note, I_*Kir*_ does not have such parameters, and was fit directly to the I-V curve (Figure 1B). From these data points, the MATLAB (The MathWorks, Natick MA) “fmincon” least squares error optimization programming algorithm was used to fit hCIC ion channel data. All relevant equations for the development of this model are listed in Supplementary Table 1.

To simulate the non-excitable hMSC and CF models, the previously developed Mayourian et al. (2016) and Maccannell et al. (2007) models were used, respectively. Both the hMSC and CF models were developed using a similar method described herein, except with different ion channels. The hMSC active channels include the calcium activated potassium, delayed rectifier-like, L-type calcium, TTX-sensitive sodium current, and transient outward currents (Mayourian et al., 2016). The CF active currents include the delayed rectifier potassium, inward rectifier potassium, and sodium-potassium pump currents (Maccannell et al., 2007). To simulate CCs, we fused hCICs and hMSCs 1:1 with a membrane capacitance set to the average of the two cell types (to be within range of experimental data) (Quijada et al., 2015; Firouzi et al., 2020), and channel currents representing the sum of each cell type, using methods previously described (Mayourian et al., 2016).

### Simulating Heterocellular Coupling Between Cardiomyocytes and Non-excitable Cells

In this study, established mathematical models of rat cardiomyocyte (Devenyi and Sobie, 2016), mouse cardiomyocyte (Bondarenko et al., 2004), healthy endocardial hCM (O’Hara et al., 2011; Mora et al., 2018), heart failure human cardiomyocyte in non-fibrotic myocardium (HF-hCM) (Mora et al., 2018), or heart failure human cardiomyocyte in fibrotic myocardium (FHF-hCM) (Mora et al., 2018) were used to model various species and disease states of cardiomyocytes. Analogous to methods previously published (Xie et al., 2009), each cell (i.e., cardiomyocyte, CF, hCIC, or hMSC) was coupled to neighboring cells using the following governing equation:


(2) $$-\text{C}\,\frac{\text{dV}}{\text{dt}}={\text{I}}_{\text{ion}}+\sum_{\text{k}=1}^{\text{n}}{\text{G}}_{\mathrm{gap},k}\,\left(V-{\text{V}}_{\text{k}}\right)$$

where C, V, I_ion_ represent the membrane capacitance, voltage (a function of time), and total current (a function of voltage and time), respectively, of a given cell type; t is time; n is the integer number of coupled neighbors (cardiomyocytes were permitted to be coupled to CFs, hCICs, hMSCs, or other cardiomyocytes; CFs were permitted to be coupled to cardiomyocytes or other CFs; hCICs were permitted to be coupled to cardiomyocytes or other hCICs; hMSCs were permitted to be coupled to cardiomyocytes or other hMSCs); G_*gap*,k_ is the gap junctional conductance between a given cell and its k^th^ neighbor; and V_*k*_ is the membrane voltage of the k^th^ neighbor.

Adapted from Mora et al. (2018), for the FHF-hCM model, 5 CFs were coupled to one HF-hCM with a gap junctional conductance of 1 nS. When incorporating the hMSC paracrine signaling variable and its anti-fibrotic effects (see section “Modeling Paracrine Signaling Effects” below for details), the corrected number of coupled CFs was rounded to the nearest integer. The only exception to this was in the section, “Role of Human Mesenchymal Stem Cell Paracrine Signaling for Correcting Heart Failure Cardiomyocytes in Fibrotic Myocardium”, where we allowed the hMSC paracrine signaling variable to be continuous in order to investigate hMSC paracrine signaling over a wide range of treatment dosages. The resultant anti-fibrotic effects of this continuous variable also made the corrected number of CFs a continuous variable. Therefore, in this case, Equation 2 for cardiomyocytes was simplified from a summation over n fibroblasts to a multiplicative factor, n_*f*_.


(3) $$-\text{C}\,\frac{\text{dV}}{\text{dt}}={\text{I}}_{\text{ion}}+{\text{n}}_{\text{f}}\,{\text{G}}_{\mathrm{gap},f}\,\left(V-{\text{V}}_{\text{f}}\right)$$

where n_f_, G_gap,f_, and V_f_ correspond to number of fibroblasts, gap junctional conductance between a fibroblast and cardiomyocyte, and fibroblast membrane potential, respectively.

### Modeling Paracrine Signaling Effects

Similar to our previous work (Mayourian et al., 2018a), a sigmoidal dose-response curve was used to characterize paracrine effects on sarcoendoplasmic reticulum calcium-ATPase and L-type calcium channel:


(4) $${\text{I}}_{\text{CaL}}^{{}^{\prime }}={\text{I}}_{\text{CaL}}\,\left(1+\frac{\Delta \,{\text{I}}_{\text{CaL}}}{1+10^{{\text{k}}_{\text{CaL}}\,\left({\text{EC}}_{50,\mathrm{CaL}}-x\right)}}\right)$$

(5) $${\text{J}}_{\text{up}}^{{}^{\prime }}={\text{J}}_{\text{up}}\,\left(1+\frac{\Delta \,{\text{J}}_{\text{up}}}{1+10^{{\text{k}}_{\text{Jup}}\,\left({\text{EC}}_{50,\mathrm{Jup}}-x\right)}}\right)$$

where I’_*CaL*_ and J’_*up*_ represent the fold changes of L-type calcium channel (I_*CaL*_) and SERCA calcium uptake activity (J_*up*_) based on prescribed hMSC paracrine signaling dosages, respectively; ΔI_*CaL*_ and ΔJ_*up*_ are the maximum saturated effects of hMSC paracrine signaling on I_*CaL*_ and J_*up*_, respectively; k_*CaL*_ and k_*Jup*_ are the characteristic Hill coefficients for each respective curve; EC_50,*CaL*_ and E_*C*50,*Jup*_ are the characteristic half maximum effective concentrations for each respective curve; and x is the effective hMSC paracrine signaling dosage, defined as Log_10_ of the percentage of hMSCs per myocyte. Parameter values were obtained by experimental calibration methods as described elsewhere (Mayourian et al., 2018a). Parameter values are shown in Supplementary Table 2.

In addition, paracrine anti-fibrotic effects of hMSCs were based on our previously determined, data-driven correlation function (y = 0.94x) between number of hMSCs delivered (x; % of total left ventricular cell population) and percentage decrease of fibrosis (y) (Mayourian et al., 2017). Based on this equation, we removed a defined percentage of fibroblasts (rounded to the nearest whole number, except in Equation 3).

Paracrine effects of hCICs on cardiomyocyte electrophysiology and calcium cycling were assumed to be negligible based on findings from Smit et al. (2017) and others (Gray et al., 2015; Agarwal et al., 2017).

### Parameter Sensitivity Analysis

To identify non-excitable cell ion channels responsible for influencing the action potential waveform, calcium transient, and net ionic channel charge of coupled HF-hCMs, we examined the model parameter sensitivity using an established multivariable regression analysis (Mayourian et al., 2016, 2017; Mora et al., 2018). More specifically, the prescribed hCIC, hMSC, and CF ion channel parameters of interest were randomly varied for 300 trials by a normally distributed pseudorandom scale factor with a coefficient of variation of 20%. From the changes in certain model outputs (i.e., action potential, calcium transient, and total ionic flux parameters of the coupled HF-hCM) resulting from the given input parameter perturbations, a linear approximation was made to find the normalized parameter sensitivity vector. Results were analyzed by hierarchical clustering and expressed as a heatmap matrix with input-parameter columns and output-sensitivity-vector rows.

### Generating Populations of Heart Failure Human Cardiomyocyte in Non-fibrotic and Fibrotic Myocardium Models

To find which combinations of hCIC and hMSC HC and PS effects might restore the function of simulated HF-hCM and FHF-hCM to healthy hCM conditions, we generated a population of 2,500 HF-hCM and 2,500 FHF-hCM models, each with variable hCIC and hMSC intervention, by randomly assigning empirically relevant parameter values to the number of hCICs per myocyte (n_*hCIC*_), number of hMSCs per myocyte (n_*hMSC*_), hCIC gap junctional conductance (G_*gap*,hCIC_), hMSC gap junctional conductance (G_*gap,hMSC*_), and the paracrine effects of n hMSCs per myocyte (n_*hMSC,PS*_). Bounding values and justifications for each parameter are given in Supplementary Table 3.

Our model calibration algorithm determined whether a given set of model parameters for the non-excitable cell-coupled HF-hCMs or FHF-hCMs should be added to the “corrected” population based on criterion that the simulated output metrics—specifically, APD at 90% repolarization (APD_90_), APD at 50% repolarization (APD_50_), peak calcium transient amplitude ([Ca^2+^]_*i*,max_), and calcium relaxation time constant (τ_ca_)–all fall within an accepted range from a healthy hCM. Modified from Mora et al. (2018), bounds on the allowed deviation from healthy hCM values for each metric were set to ± 50% of the disease disparity as follows:


(6) metric±healthy0.5(metric-healthymetric)failing

where “metric” represents APD_90_, APD_50_, [Ca^2+^]_*i*,max_, τ_ca_. Parameter sets were considered “corrected” if they satisfied this acceptance criterion for all four metrics.

## Results

### Human c-Kit^+^ Cardiac Interstitial Cell Electrophysiology Model Development and Validation

To investigate the effects of hCICs on cardiomyocytes, we developed a novel computational model of hCIC electrophysiology. To do so, established equations that capture gating kinetics and maximal fluxes for individual membrane channels were fit to experimental patch-clamp studies (Zhang et al., 2014) of I_*KCa*_, I_*Kir*_, I_*to*_, and I_*Na*_ activity in cultured hCICs (see Supplementary Figures 1–3 for digitized experimental data and model fits).

Based on these building blocks for the mathematical model, Figures 1A–D show the simulated I-V relationships for each respective channel in comparison to its experimental counterpart. Figures 1E–H show a simulated voltage-clamp experiment for each channel (voltage-clamp protocol inset to the right, corresponding to Zhang et al., 2014 experimental protocols). Note the sodium channel I-V curve (Figure 1C) and simulated voltage-clamp experiment (Figure 1G) have different voltage protocols in order to match experimental conditions from Zhang and coworkers. For each ion channel, the voltage-clamp model simulation (Figures 1E–H) was representative of its published experimental counterpart (Zhang et al., 2014). This provides an initial validation of our mathematical model since (except for Figure 1B) these I-V relationships were not fit directly but were simulated by the model, based on the fitted activation/inactivation data in Supplementary Figures 1–3. A shift of ∼10 mV was noted in the middle region of the I_*Na*_ curve (Figure 1C); however, as explained in the discussion, we believe this negligibly impacts the following results and conclusions of this study. Interestingly, as in the patch clamp experiments (Zhang et al., 2014), I_*Na*_ and I_*to*_ had steady-state non-inactivating currents at the end of the voltage clamp, similar to I_*to*_ in hMSCs (Li et al., 2005). Further, we note the difference between sodium currents in hCICs and cardiomyocytes—I_*Na*_ in hCIC is mainly encoded by Nav1.3 and Nav1.6, and is very sensitive to inhibition by TTX in the nanomolar range, whereas human cardiac myocyte I_*Na*_ (Nav1.5) is usually blocked by TTX in the micromolar range (Zhang et al., 2014).

**FIGURE 2:**
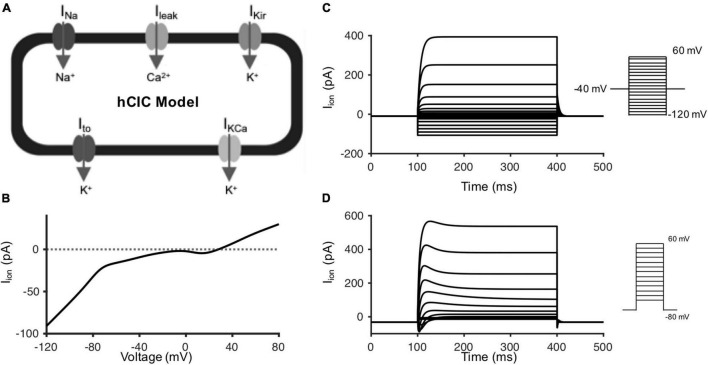
Whole-cell hCIC electrophysiology model. **(A)** Schematic of hCIC electrophysiology model incorporating the large-conductance Ca^2+^-activated K^+^ channel (I_*KCa*_), inward rectifying K^+^ channel (I_*Kir*_), Na^+^ channel (I_*Na*_), transient outward channel (I_*to*_), and leakage (I_*leak*_) currents. **(B)** Simulated current-voltage (I-V) relationship curve of the whole-cell hCIC model activated by a voltage ramp (−120 to + 80 mV over 3 s) from a holding potential of −40 mV, analogous to the experimental setup from Figure 2E of Zhang et al. (2014). Dotted line added as reference for net zero current. **(C)** Voltage-clamp simulation (voltage step protocol inset) for hCIC cell with I_*KCa*_ and I_*Kir*_ functional channels, corresponding to Figure 2D of Zhang et al. (2014). **(D)** Voltage-clamp simulation (voltage step protocol inset) for hCIC whole-cell electrophysiology model, representing the average of 3 individual hCICs from Figures 2A–C of Zhang et al. (2014).

Building on this foundation of individual hCIC ion channel models, a whole-cell model incorporating I_*KCa*_, I_*Kir*_, I_*Na*_, and I_*to*_ was established (Figure 2A); in addition, a leakage current was added to set the resting membrane potential at −32.5 mV, equivalent to the mean experimental value (Zhang et al., 2014). Figure 2B shows the current-voltage relationship during a voltage ramp simulation from −120 to + 80 mV of the whole-cell hCIC mathematical model, which is representative of the corresponding published experiment (Figure 2E of Zhang et al., 2014). As expected, the previously acknowledged ∼10 mV shift noted above for the I_*Na*_ curve (Figure 1C) is also apparent in the overall voltage ramp simulation (Figure 1E). While the current magnitudes are similar between the simulated and experimental data within physiologic ranges of cardiomyocyte voltages (i.e., −85 and 40 mV), we note that outside of these physiologic ranges (i.e., < −85 mV and > 40 mV) our magnitudes are lesser in the simulations, which may be explained by our simulations representing mean data rather than a single cell (as in Figure 2E of Zhang et al., 2014). Voltage-clamp simulations of hCICs in which only the I_*KCa*_ and I_*Kir*_ channels are functional (Figure 2C) are also representative of their experimental counterpart (Figure 2D of Zhang et al., 2014). Finally, voltage-clamp simulations of a whole-cell hCIC (Figure 2D) are representative of the average of three individual hCICs, each expressing variable functional channels (Figures 2A–C of Zhang et al., 2014). Altogether, having multiple examples validating our whole-cell model with published experimental data provided the justification to couple this hCIC model to cardiomyocytes to study the resulting electrophysiological effects.

### Coupling Human c-Kit^+^ Cardiac Interstitial Cell With Multiple Cardiomyocyte Species

**FIGURE 3:**
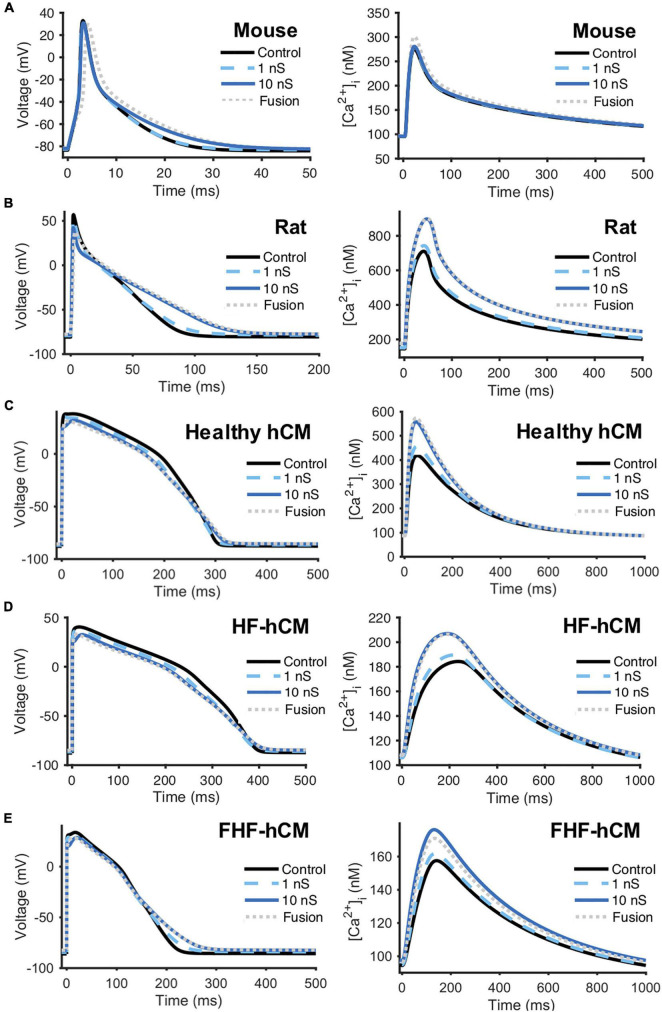
hCIC heterocellular coupling effects on multi-species cardiomyocyte action potential and calcium transient. Simulation of heterocellular coupling between hCICs and **(A)** adult mouse ventricular cardiomyocytes, **(B)** adult rat ventricular cardiomyocytes, **(C)** healthy adult human ventricular cardiomyocytes (hCM), **(D)** human adult heart failure ventricular cardiomyocytes in non-fibrotic myocardium (HF-hCM), and **(E)** human adult heart failure ventricular cardiomyocytes in fibrotic myocardium (FHF-hCM). Resultant cardiomyocyte action potential (left) and calcium transient (right) are shown. Simulations assumed 1:1 hCIC:cardiomyocyte coupling under 1 nS (dashed cyan), 10 nS (solid navy), and fusion (dotted gray) conditions, compared to cardiomyocyte-only controls (solid black). Note the shorter time axis used for mouse and rat cells compared to that for human cells.

As an initial step toward modeling hCICs for cardiotherapeutics, we started with the simple case of coupling of hCICs to individual cardiomyocytes from multiple relevant species, including mouse cardiomyocytes (Bondarenko et al., 2004; Figure 3A), rat cardiomyocytes (Devenyi and Sobie, 2016; Figure 3B), healthy hCM (O’Hara et al., 2011; Mora et al., 2018; Figure 3C), HF-hCM (Mora et al., 2018; Figure 3D), and FHF-hCM (Mora et al., 2018; Figure 3E). Gap junctional conductance mediated largely via connexin-43 (Dergilev et al., 2018) was modeled as physiologically low (1 nS), high (10 nS), or sufficiently high to approximate cell-cell fusion (Valiunas et al., 2004). In these simulations, a 1:1 ratio of hCIC:cardiomyocyte was assumed.

Detailed effects of hCIC HC on the cardiomyocyte action potential and calcium transient waveforms varied by species and cell phenotype (Figure 3). Nonetheless, across all species, treating myocytes with hCIC HC decreased upstroke velocity (UV), decreased peak action potential voltage (V_peak_), depolarized RMP, and increased [Ca^2+^]_*i*,max_ relative to untreated controls; the effects increased with gap junction conductance from 1 to 10 nS, with minimal further changes due to cell fusion. Consistent with *in vitro* experimental work (Smit et al., 2017), simulated treatment of rat cardiomyocytes with hCIC HC led to prolonged APD, depolarized RMP, and decreased UV when compared to control untreated rat cardiomyocytes (Figure 3B). As anecdotally noted in the same study (Smit et al., 2017), we also predicted V_peak_ to be decreased when treating with hCICs. Unlike with rat cardiomyocytes, hCIC coupled to mouse cardiomyocytes were predicted to have minimal effects on action potential and calcium transient (Figure 3A), which may partly reflect the ∼50% larger membrane capacitance of the mouse cardiomyocyte model. For the human myocyte models, hCIC HC reduced UV and V_peak_ across healthy and diseased hCM phenotypes, and also shortened APD_50_ while lengthening APD_90_, thus prolonging APD triangulation (Figures 3C–E). Although hCIC HC also increased [Ca^2+^]_*i*,max_ in healthy and diseased coupled hCMs, the simulations revealed a monotonic relationship between [Ca^2+^]_*i*,max_ and G_*gap*_ with healthy hCMs (Figure 3D), in contrast to a non-monotonic relationship in the case of HF-hCMs and FHF-hCMs (Figures 3D,E), where [Ca^2+^]_*i*,max_ increased with G_*gap*_ from 1 to 10 nS, but then decreased slightly from 10 nS to fusion conditions in the diseased hCMs.

### Comparison of Human c-Kit^+^ Cardiac Interstitial Cell and Human Mesenchymal Stem Cell Effects on Human Ventricular Cardiomyocytes

**FIGURE 4:**
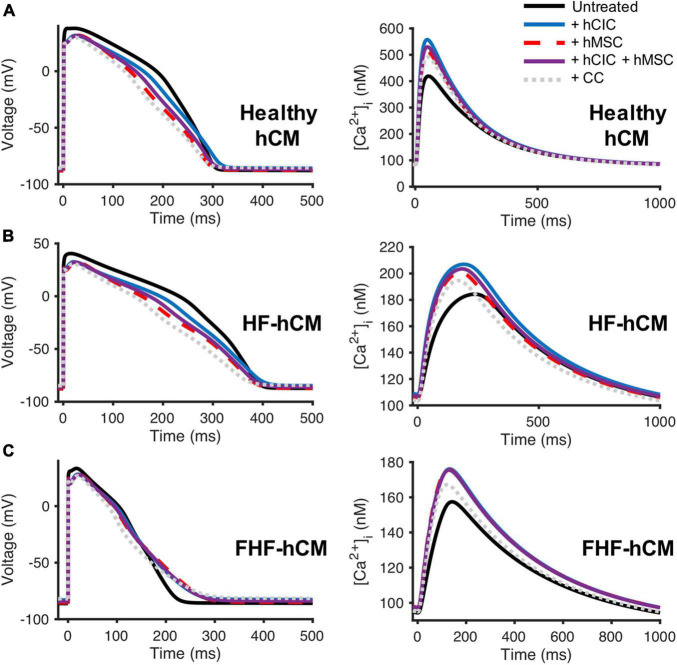
Comparison of hCIC and hMSC heterocellular coupling effects on human ventricular cardiomyocyte action potential and calcium transient. Simulation of hCIC and hMSC heterocellular coupling effects on **(A)** healthy adult human ventricular cardiomyocytes (hCM), **(B)** human adult heart failure ventricular cardiomyocytes in non-fibrotic myocardium (HF-hCM), and **(C)** human adult heart failure ventricular cardiomyocytes in fibrotic myocardium (FHF-hCM), examining the resulting cardiomyocyte action potential (left) and calcium transient (right). Simulations of a supplemented myocyte coupled at 10 nS as follows: 1:1 to hCIC (+hCIC; blue); 1:1 to hMSC (+hMSC; dashed red); 1:1 to a combination of half hCIC plus half hMSC (+hCIC +hMSC; solid purple); or 1:1 to fused hCIC-hMSC CardioChimera (+CC; dotted gray), compared to untreated cardiomyocyte-only controls (solid black).

Next, we aimed to evaluate the HC effects of hCICs in the context of other non-excitable cells; for example, combined hCIC and hMSC cardiotherapy is relevant to the CONCERT-HF clinical trial (Bolli et al., 2021). Therefore, we simulated untreated (control) vs. treated healthy and diseased hCMs coupled 1:1 to hCIC (+hCIC), 1:1 to hMSC (+hMSC), 1:1 to a combination of half hCIC plus half hMSC (+hCIC +hMSC), or 1:1 to a fused hCIC-hMSC CardioChimera (+CC) (Quijada et al., 2015), all with a G_*gap*_ of 10 nS. The myocytes examined included adult healthy hCM as well as failing HF-hCMs or FHF-hCMs. In general, the addition of hCICs and hMSCs: (1) shortened APD_50_; (2) increased [Ca^2+^]_*i*,max_; and (3) depolarized RMP (Figure 4). These trends also held true when other G_*gap*_ values were tested (data not shown).

In comparison to conditions involving hMSC coupling (i.e., +hMSC, +hCIC +hMSC, and +CC), hCIC-only HC (+hCIC) led to the least variation compared to the control adult hCM action potential waveform. However, for both healthy hCMs and HF-hCMs, +hCIC increased [Ca^2+^]_*i*,max_ more than any hMSC-mediated coupling. In FHF-hCMs (Figure 4C), the effects of supplementing with hCIC HC were nearly indistinguishable from supplementing with hMSC HC for both the action potential and the calcium transient. Adding non-excitable cells to cardiomyocytes 1:1 comprised of half-hMSC and half-hCIC (i.e., +hCIC +hMSC) was intermediate between +hMSC and +hCIC (Figure 4), absent of any notable synergistic HC effects when combining the two cell types. Finally, +CC effects on healthy and diseased hCM action potential were more pronounced, with lower [Ca^2+^]_*i*,max_ than +hCIC, +hMSC, and +hCIC +hMSC. While HC of hMSCs and hCICs increased the [Ca^2+^]_*i*,max_ of failing hCMs (Figures 4B,C), the effects did not appear sufficient to restore calcium transients back to the level of healthy hCMs (Figure 4A). Again, these trends held true for other values of G_*gap*_ tested (data not shown).

### Comparison of Non-excitable Cell Sinking Effects on Heart Failure Cardiomyocytes

**FIGURE 5:**
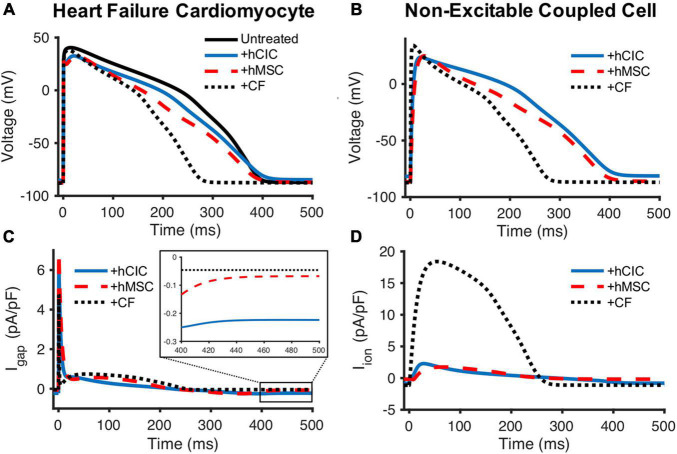
Comparison of hCIC, hMSC, and CF sinking effects on HF-hCMs. **(A)** Heart failure cardiomyocyte (HF-hCM) action potential when coupled to one hCIC (+hCIC, solid blue), one hMSC (+hMSC, dashed red), or one cardiac fibroblast (+CF, dotted black), with a gap junctional conductance of 10 nS, compared to untreated control HF-hCM (solid black). **(B)** Transmembrane potential of the respective coupled non-excitable cell. **(C)** Gap junction current density (I_*gap*_) between the HF-hCM and respective coupled non-excitable cell; positive I_*gap*_ corresponds to positive flow out of the myocyte and into the coupled non-excitable cell. **(D)** Total ionic current density of the coupled non-excitable cell.

Next, we aimed to gain insight into the mechanisms underlying the simulated, non-excitable cell-dependent HC effects on cardiomyocyte action potential and calcium transient. To do so, we first explored the general electrical source and sink behavior during HC at 10 nS between one HF-hCM and either one hCIC, one hMSC, or one CF (Figure 5).

The CF acts as a sink during upstroke and plateau phases of the cardiomyocyte action potential (Figure 5A) with a net positive ionic flow from the HF-hCM to the CF (Figure 5C). By comparison, the hMSC had less of a sink effect, and the hCIC had the lowest magnitude sink effect during HF-hCM upstroke and plateau despite their larger capacitances than CFs; accordingly, this corresponded to substantially lower magnitude total outward ionic currents for these non-excitable cells in comparison to CFs (Figure 5D). hCIC served as the largest electrical source (i.e., negative I_*gap*_, indicating net positive flow from the non-excitable cell to the HF-hCM) during repolarization (Figure 5C). This persisted during the HF-hCM’s resting phase due to the hCIC I_*Kir*_, such that hCIC coupling resulted in a more depolarized intrinsic HF-hCM RMP relative to the other non-excitable cell types. Given the prescribed gap junctional conductance of 10 nS, the transmembrane potential of the non-excitable coupled cell (Figure 5B) appeared nearly identical to the coupled HF-hCM (Figure 5A).

### Parameter Sensitivity Analysis to Study Mechanisms of Non-excitable Cell Effects on Action Potentials and Calcium Transients in Coupled Cardiomyocytes

**FIGURE 6:**
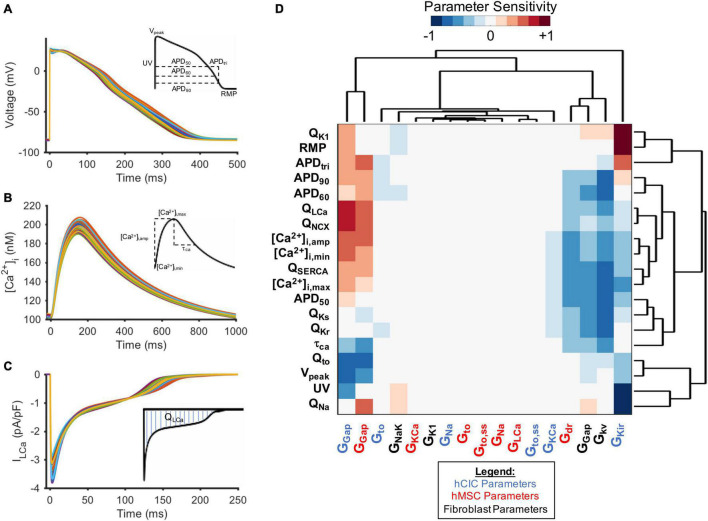
Parameter sensitivity analysis identifies key non-excitable cell ion channels modulating heart failure myocyte action potential and calcium transient metrics. Randomly perturbing input parameters of interest for 300 trials led to a range of **(A)** action potentials, **(B)** calcium transients, and **(C)** ionic current waveforms (L-type calcium channel current, I_*LCa*_, shown as representative example) for HF-hCMs. Output metrics measured for each waveform are inset. **(D)** Hierarchical clustering of heatmap matrix with input non-excitable cell ionic channel maximal conductance columns and output parameter sensitivity value rows. Non-standard abbreviations: Net charge for ionic current x (Q_x_); action potential duration at x% repolarization (APD_x_); APD triangulation (APD_tri_); resting membrane potential (RMP); peak voltage (V_peak_); upstroke velocity (UV); calcium transient time constant (τ_Ca_); conductance of channel x (G_x_); calcium transient amplitude ([Ca^2+^]_i,amp_); diastolic intracellular calcium ([Ca^2+^]_*i*,min_); systolic intracellular calcium ([Ca^2+^]_*i*,max_).

To further study the ionic mechanisms underlying the simulated non-excitable cell-dependent HC effects on cardiomyocyte action potential and calcium transient, we performed a parameter sensitivity analysis. Figure 6 shows the HF-hCM action potential (Figure 6A), calcium transient (Figure 6B), and net ionic channel charge (Figure 6C) tracings for 300 simulations resulting from pseudorandom perturbations of input ion channel parameters of interest for coupled hCICs, hMSCs, and CFs. Hierarchical clustering analysis (Figure 6D) confirmed that the resulting HF-hCM output parameters SERCA and sodium-calcium exchanger net charges (Q_*SERCA*_ and Q_*NCX*_, respectively) clustered with calcium transient output parameters they are known to influence. Similarly, HF-hCM inward rectifying potassium channel net channel charge (Q_*K1*_) clustered with RMP, rapidly and slowly activating delayed rectifier potassium channel net channel charge (Q_*Kr*_ and Q_*Ks*_, respectively) clustered with APD_50_, transient outward channel net flux (Q_*to*_) clustered with V_peak_, and sodium channel net channel charge (Q_*Na*_) clustered with UV, all as would be expected based on known interdependencies.

Mechanistically, while RMP and UV were not particularly sensitive to coupled hMSC parameters (Figure 6D), they were sensitive to hCIC channel inward rectifying potassium channel maximal conductance (G_*Kir*_), supporting the mechanistic role of I_*Kir*_ in hCIC HC-induced RMP depolarization. In addition, both hCIC and hMSC gap junction conductances positively correlated with the net flux (Q) through LTCC and NCX, explaining the positive impact on other calcium cycling outputs, including calcium transient amplitude ([Ca^2+^]_i,amp_), diastolic calcium ([Ca^2+^]_*i*,min_), and peak systolic calcium ([Ca^2+^]_*i*,max_). Further exploration of the mechanism revealed that hCIC and hMSC coupling led to a lower membrane voltage during the plateau phase (Figure 4), which is in fact closer to the optimal peak I_*LCa*_ that has previously been described (O’Hara et al., 2011).

Clustering distinctly from hMSCs and hCICs, CF gap junctional conductance negatively correlated with APD outputs and calcium transient parameters (Figure 6D). Interestingly, this CF parameter, as well as the CF delayed rectifier potassium conductance (G_*kv*_), clustered with hCIC G_*Kir*_ and hMSC delayed rectifier maximal conductance (G_*dr*_), all of which negatively correlated with APD outputs and calcium transient parameters. Among all ion channel conductances, the largest magnitude negative correlation was seen for the CF delayed rectifier potassium conductance, which plays a key role in the greater total outward ionic current seen in Figure 5D, helping to explain the unique effect of CFs compared to hMSCs and hCICs in substantially decreasing APD.

### Population-Based Modeling Approach to Optimize Cell Delivery Strategies for Correcting Failing Human Cardiomyocytes

Next, the computational models were used to assess whether hMSCs and/or hCICs are theoretically capable of restoring electrical and calcium cycling behavior of failing HF-hCM and FHF-hCM back toward healthy hCMs.

**FIGURE 7:**
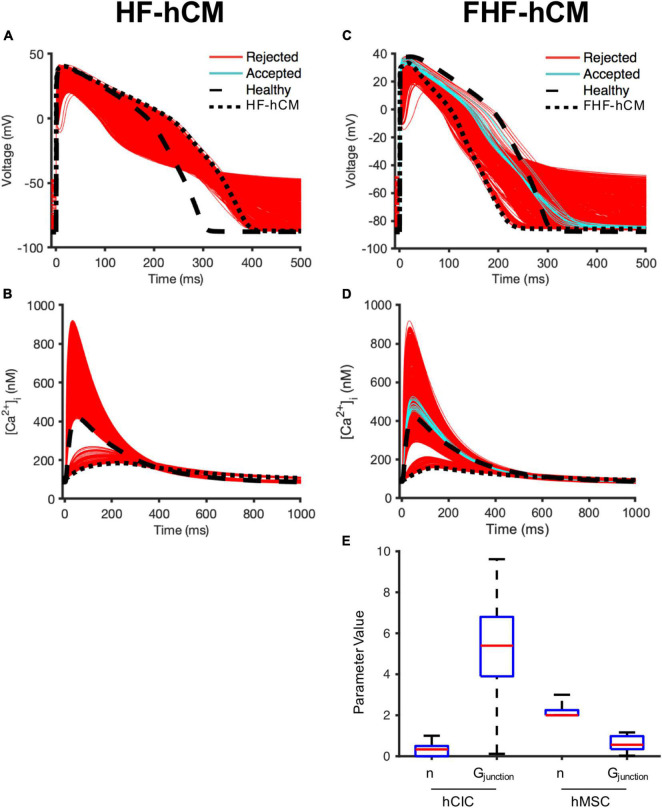
Variable cell delivery intervention effects on fibrotic and non-fibrotic heart failure cardiomyocytes. A population of 2,500 HF-hCM and FHF-hCM models with variable hCIC and hMSC intervention by randomly assigning empirically relevant parameter values to integer number of coupled hCICs and hMSCs per myocyte (n_*hCIC*_ and n_*hMSC*_, respectively), and hCIC and hMSC gap junctional conductance (G_*junction*_) leads to modification of action potential and calcium transient waveforms in the coupled HF-hCM (**A,B**, respectively) and FHF-hCM (**C,D**, respectively). Untreated healthy (control) and heart failure cardiomyocyte models are in dashed and dotted black lines, respectively. Coupled heart failure models that satisfy a prescribed ± 50% restoration criteria relative to healthy cardiomyocyte action potential and calcium transient metrics (based on APD_90_, APD_50_, [Ca^2+^]_*i*,max_, and τ_ca_) were accepted (cyan); models outside this range were rejected (red). **(E)** Boxplot representation of population of hCIC and hMSC parameters leading to accepted FHF-hCM models, showing median, 25th and 75th percentile confidence intervals, and min/max values. Values for G_*junction*_ are in units of nS.

First, we simulated the effects of variable hMSC and hCIC interventions for HF-hCM in non-fibrotic myocardium. While several of the model permutations were capable of correcting the calcium transient to near-healthy conditions (Figure 7B), none of the model permutations (0/2,500) for hMSC and/or hCIC intervention was able to satisfy the predefined criteria that APD_90_, APD_50,_ [Ca^2+^]_*i*,max_, and τ_ca_ would all be corrected to within 50% of the disease related deviation from healthy values (note absence of cyan curves in Figures 7A,B).

By contrast, approximately 1% (21/2,500) of model permutations (cyan, Figures 7C,D) satisfied the predefined criteria for acceptance when delivering hMSCs and/or hCICs to FHF-hCMs in fibrotic myocardium. Distributions of parameter values for the accepted models are presented in Figure 7E. Interestingly, unlike n_*hCIC*_, the accepted values of n_*hMSC*_ did not approach zero. In addition, accepted values of gap junctional conductance spanned essentially the full range permitted for hCICs, including nearly zero nS. While not spanning the full range of permitted values, hMSC gap junctional conductances also included values near zero nS, suggesting cardiomyocyte correction could be achievable without hCICs and without direct coupling of hMSCs.

### Role of Human Mesenchymal Stem Cell Paracrine Signaling for Correcting Heart Failure Cardiomyocytes in Fibrotic Myocardium

**FIGURE 8:**
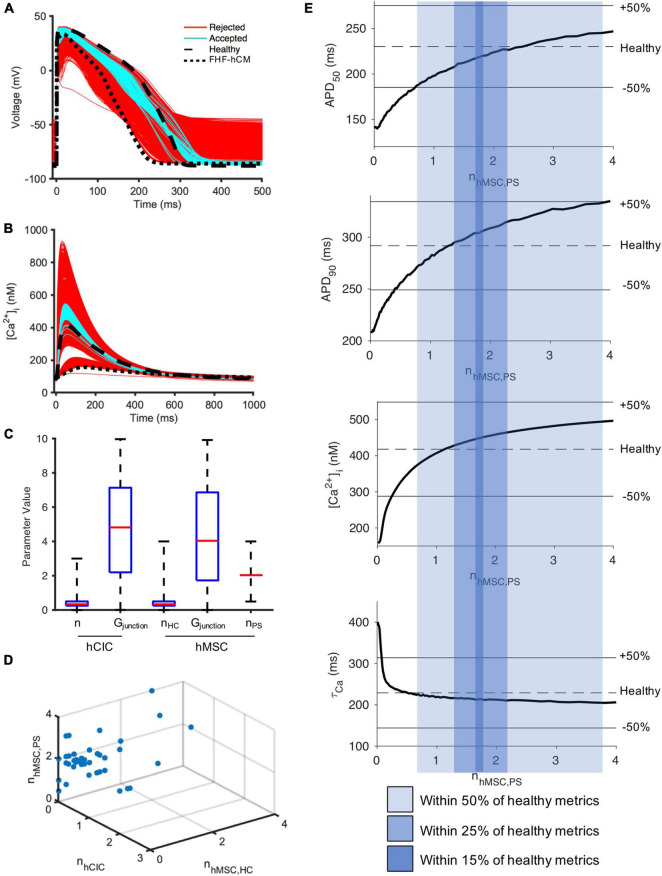
hMSC paracrine signaling for correcting fibrotic heart failure cardiomyocyte electrophysiology and calcium cycling. A population of 2,500 FHF-hCM models with variable hCIC and hMSC intervention by randomly assigning empirically relevant parameter values to integer number of coupled hCICs and hMSCs per myocyte (n_*hCIC*_ and n_*hMSC,HC*_, respectively), hCIC and hMSC gap junctional conductance (G_*junction*_) and the paracrine signaling effects of n hMSCs per myocyte (n_*hMSC,PS*_) leads to modification of **(A)** action potential and **(B)** calcium transient waveforms in the coupled FHF-hCM. Untreated healthy (control) and FHF-hCM isolated cardiomyocyte models are in dashed and dotted black lines, respectively. Stem cell-coupled heart failure models that met a predefined restoration criteria toward healthy cardiomyocytes were accepted (cyan); models outside this range were rejected (red). **(C)** Boxplot representation of population of hCIC and hMSC parameters leading to accepted models, showing median, 25th and 75th percentile confidence intervals, and min/max values. Values for G_*junction*_ are in units of nS. **(D)** Accepted set of n_*hCIC*_, n_*hMSC,HC*_, and n_*hMSC,PS*_. **(E)** Effects of PS alone (with zero HC enforced) on FHF-hCM APD_50_, APD_90_, [Ca^2+^]_*i*,max_, and τ_ca_. Upper and lower bounds with shaded regions indicating correction to within ± 50, ± 25, and ± 15% of the deviation from healthy metrics are provided as reference. Note that in **(E)**, n is treated as a non-integer.

To investigate why n_*hMSC*_ had a more narrow window of accepted values that did not include zero (Figure 7E), we repeated the FHF-hCM experiment from Figures 7C–E, but allowed hMSC HC and PS parameters to vary independently (n_*hMSC,HC*_ and n_*hMSC,PS*_, respectively). Approximately 7% (178/2,500) of model permutations (cyan, Figures 8A,B) satisfied the predefined criteria for acceptance by restoring all four action potential and calcium handling metrics to within 50% of the disease related difference from healthy values. Distributions of parameter values for the accepted models are presented in Figure 8C. Accepted values of gap junctional conductances spanned essentially the full range permitted for both hCIC- and hMSC-coupling, including zero nS. Similarly, the total number of hCICs and hMSCs coupled per FHF-hCM was highly variable, with a range that included zero. By contrast, n_*hMSC,PS*_ had a range of accepted values equivalent to approximately one-half to four stem-cells-worth of paracrine effects per myocyte. Notably, none of the models that satisfied the acceptance criteria did so with zero n_*PS*_ (Figures 8C,D), indicating that PS from hMSCs was necessary for restoration of FHF-hCM characteristics back toward healthy conditions. In addition, the fact that acceptable models were found that included PS alone without hCIC or hMSC HC (i.e., n_*PS*_ ≠ 0 with n_*hCIC*_ = n_*hMSC*_ = 0; Figure 8D) indicates that the effects of hMSC PS alone may be sufficient to restore failing cardiomyocyte waveforms to nearly healthy cardiomyocyte conditions.

Therefore, we studied the effects of hMSC paracrine signaling in isolation. Indeed, when the condition of zero HC was enforced, Figure 8E shows that 0.7–3.8 hMSC worth of PS effects per myocyte alone were sufficient to satisfy the 50% acceptance criteria. In fact, n_*hMSC*,*PS*_ values in the range 1.3–2.2 could satisfy a more stringent acceptance criteria of 25% deviation from healthy metrics; and remarkably, PS effects alone were theoretically sufficient to get as close as within 15% of healthy conditions when n_*hMSC,PS*_ values in the range 1.7–1.8 (Figure 8E).

### Examining Potential Risks of Non-excitable Cell Supplementation

As with every therapeutic assessment, one must strive to not only maximize the benefits but also minimize potential adverse effects. Therefore, given the potential pro-arrhythmic risks previously posed for cell-based therapeutics (Chang et al., 2006), we evaluated single-cell indicators of tissue-level conduction velocity and arrythmogenicity—namely RMP and UV.

**FIGURE 9:**
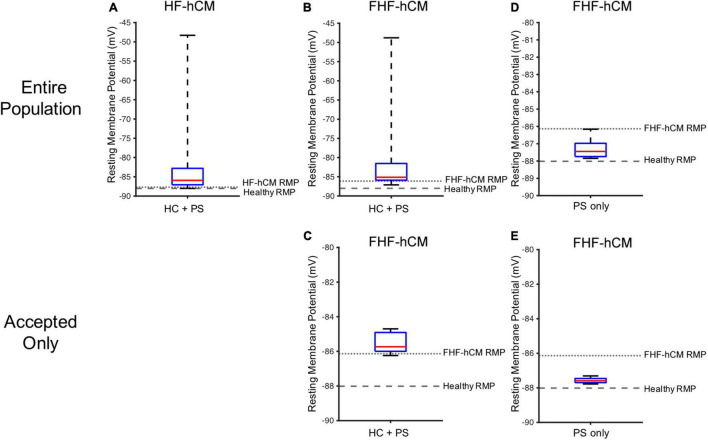
Variable cell delivery intervention effects on heart failure cardiomyocyte resting membrane potential. Simulated effects of entire 2,500 population of variable hCIC and hMSC HC and PS intervention on **(A)** HF-hCM and **(B)** FHF-hCM resting membrane potential (RMP). **(C)** Simulated effects of accepted population of variable hCIC and hMSC HC and PS intervention on FHF-hCM RMP. **(D,E)** Simulated effects of **(D)** entire 2,500 population and **(E)** accepted only population of variable hMSC PS only intervention on FHF-hCM RMP. Note expanded *y*-axis range for **(A,B)**.

As shown in Figure 9, the simulated RMP for HF-hCM subjected to variable hCIC and hMSC interventions (using all 2,500 simulations from Figures 7A,B) tended to increase RMP above the uncoupled cell values, with some instances depolarized beyond −50 mV (Figure 9A). The simulated RMP for FHF-hCM subjected to variable hCIC and hMSC interventions (using all 2,500 simulations from Figures 7C,D) similarly exacerbated the increases in RMP associated with the fibrotic heart failure model (Figure 9B). When considering only that population of models that met our acceptance criteria (i.e., accepted cyan curves in Figures 7C,D), the range and maximal RMP reduced drastically, with none worse than −84 mV (Figure 9C), but the RMP values still remained no better than the uncoupled FHF-hCM model. However, for variable hMSC paracrine signaling interventions alone on FHF-hCM (i.e., the entire population from Figure 8E), we see that paracrine signaling alone leads not only to a small range of RMP, but also closer to healthy levels (Figure 9D). Restoration toward healthy conditions is further improved (Figure 9E) by only using accepted paracrine signaling interventions alone on FHF-hCM (i.e., the accepted population from Figure 8E).

**FIGURE 10:**
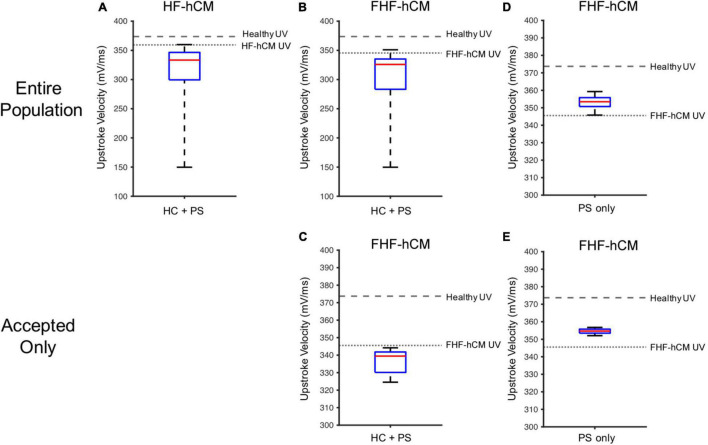
Variable cell delivery intervention effects on heart failure cardiomyocyte upstroke velocity. Simulated effects of entire 2,500 population of variable hCIC and hMSC HC and PS intervention on **(A)** HF-hCM and **(B)** FHF-hCM upstroke velocity (UV). **(C)** Simulated effects of accepted population of variable hCIC and hMSC HC and PS intervention on FHF-hCM UV. **(D,E)** Simulated effects of **(D)** entire 2,500 population and **(E)** accepted only population of variable hMSC PS only intervention on FHF-hCM UV. Note expanded *y*-axis range for **(A,B)**.

Similar trends hold for UV (Figure 10), such that unfiltered non-excitable cell supplementation decreases UV drastically by greater than 50% (Figures 10A,B), filtering according to the acceptance criteria leads to a narrower UV range (Figure 10C), and paracrine signaling alone leads to an even narrower range that approaches healthy values (Figures 10D,E).

## Discussion

**FIGURE 11:**
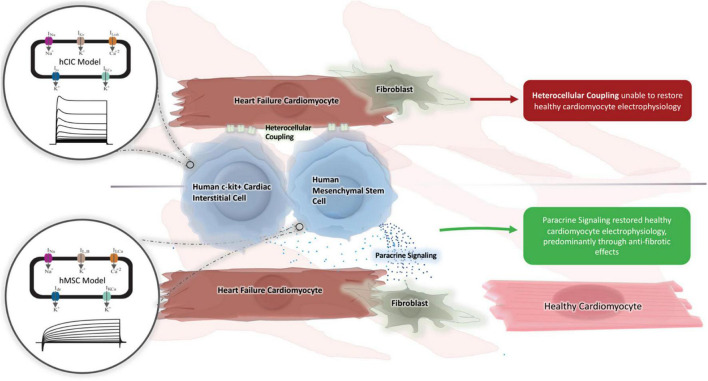
Graphical summary. A novel computational model for hCIC electrophysiology was created and validated against existing animal models. We subsequently tested whether hMSC and hCIC HC and PS were capable of restoring HF-hCM and FHF-hCM action potential and calcium handling toward those for healthy cardiomyocytes. Only hMSC models were able to restore FHF-hCM electrophysiology, and paracrine signaling was identified as necessary and sufficient for this restoration.

Cell-based strategies for treating heart failure have shown promise (Sanganalmath and Bolli, 2013; Mathiasen et al., 2019), but realizing their therapeutic potential requires a better understanding of the mechanisms underlying their interaction with host myocardium. Therefore, in this study, we (1) developed and validated a new mathematical model of hCIC electrophysiology, (2) used the model to identify the key determinants of HC effects on the action potential and calcium transient of healthy and failing hCMs, and (3) demonstrated a computational approach for estimating the potential for HC and PS effects of hCICs and hMSCs to correct the abnormal electrical and calcium cycling properties of heart failure in fibrotic and non-fibrotic myocardium while minimizing adverse pro-arrhythmic effects, providing a rationale on which to base future efforts to improve cell therapies (Figure 11).

### Utility of *in silico* Approach to Study the Non-excitable Cell-Cardiomyocyte Interactome

The non-excitable cell-cardiomyocyte interactome in this study is complex and difficult to elucidate experimentally; alternatively, computational methods offer a convenient, inexpensive, and practical tool to investigate such heterocellular interactions. To our knowledge, no experimental electrophysiology studies have been conducted using hCIC- and/or hMSC-coupled adult hCMs, reflecting the difficulties accessing primary hCMs and their limited capacity for *in vitro* cell culture. While fibroblast-myocyte HC has been well studied computationally (Maccannell et al., 2007; Sachse et al., 2009; Xie et al., 2009), our group is (to our knowledge) the first and only to develop hMSC (Mayourian et al., 2016) and hCIC (this study) electrophysiology models and to computationally investigate their HC effects when coupled to cardiomyocytes.

Herein, we perform several *in silico* experiments to gain new mechanistic insights into the effects of non-excitable cells on cardiomyocytes, including: (1) comparing the relative effects of hCICs to other clinically relevant non-excitable cells; (2) perturbing parameters in the non-excitable cell models for a parameter sensitivity analysis to identify key ion channels contributing to the cardiomyocyte response to each non-excitable cell type; and (3) performing a 2,500-model population-based study of electrophysiological and calcium handling effects of variable hCIC and hMSC interventions to identify paracrine signaling as a necessary and sufficient mechanism for correcting the abnormal action potential and calcium transient in failing cardiomyocytes, predominantly through anti-fibrotic mechanisms, while also minimizing pro-arrhythmic risk factors.

### Validation of Human c-Kit^+^ Cardiac Interstitial Cell Electrophysiology Model

Our hCIC model was designed to fit activation and inactivation parameters of individual channels (Supplementary Figures 1–3), and the resulting I-V and voltage clamp simulations were generally consistent with experimental characterization of ion channels in cultured c-kit^+^ cells derived from human atrial specimens (Zhang et al., 2014). One exception was a ∼10 mV shift between the experimental points and the simulated I-V curve for the sodium channel (Figure 1C), which reflects the fact that the model was fit to the constitutent activation and inactivation parameters (Supplementary Figure 1) rather than fitting the I-V curve directly. Thus, the I-V curve comparisons in Figure 1 are one form of model validation, and despite this discrepancy, the model matched the remainder of the I-V relationship for I_*Na*_ (and the other channels) reasonably well. Importantly, the sensitivity analysis showed that the I_*Na*_ component of the hCIC model has minimal effect on all cardiomyocyte action potential characteristics, so the noted discrepancy would not impact the overall results and conclusions of this study. This discrepancy corresponded to the same ∼10 mV shift noted in the voltage ramp simulation. Of note, while the current magnitudes are similar between simulated and experimental data within physiologic ranges of cardiomyocyte voltages, our magnitudes are lesser than experimental data outside of these physiologic ranges. This may be explained by our simulations representing mean data rather than a single cell (as in Figure 2E of Zhang et al., 2014), again having no consequence on the effects on cardiomyocytes as the simulated magnitude is representative of experimental data within the range of action potential voltages.

As a next step in validating the new hCIC model, we confirmed that our simulations (Figure 2) are representative of families of currents measured experimentally (Zhang et al., 2014). Furthermore, our hCIC model was consistent with the few published experiments that coupled c-kit^+^ cells with non-human species of cardiomyocytes. In particular, Smit et al. (2017) observed that HC of hCICs to rat ventricular cardiomyocytes resulted in a depolarized RMP and decreased UV as well as prolonged APD compared to uncoupled rat cardiomyocyte controls. Anecdotally, they also noted decreased peak voltages in coupled cells compared to uncoupled controls. Similar trends were reproduced by our simulated HC of hCICs and rat cardiomyocytes (Figure 3B). Additionally, Tufan et al. (2012) reported that connexin-43-mediated HC of engineered mouse CICs and neonatal rat cardiomyocytes resulted in decreased density of cardiomyocyte inward sodium currents, observations which are consistent with our model-predicted decrease in UV.

Finally, as described in the subsequent section, our simulations also provide insights that complement findings from translational studies, and further support several ongoing efforts to improve cell-based heart failure therapies.

### Interpretation and Clinical Significance of Findings

Emerging hCIC- and hMSC-based therapies represent promising treatments that are still being actively developed (Sanganalmath and Bolli, 2013; Mathiasen et al., 2019). In particular, hCIC delivery has reportedly improved ventricular function in pre-clinical studies (Hong et al., 2014; Tang et al., 2016), via mechanisms other than the previously proposed differentiation of hCICs into *de novo* cardiomyocytes (Van Berlo et al., 2014). Similar promise has been shown for hMSCs (Hare et al., 2012; Heldman et al., 2014; Karantalis et al., 2014), as well as combinations of the two cell types (Williams et al., 2013), and even hybrid stem cells created by fusing hCICs and hMSCs prior to delivery (Quijada et al., 2015). Enthusiasm from the scientific community rapidly propelled such cell-based therapies to clinical trials, and despite highly publicized setbacks, at least one randomized, double-blind, placebo-controlled phase II trial testing the combination of hMSCs and hCICs (i.e., CONCERT-HF) has recently completed (Bolli et al., 2021). Such activities underscore the value in developing a rational approach to optimize cell-based cardiotherapeutic strategies. In our study, we provide insights from *in silico* models that complement recent translational studies to offer such a rational approach for improving future cell therapies for heart failure.

To do so, we simulated the cardiomyocyte response to heterocellular coupling with translationally relevant non-excitable cells—namely hCICs, hMSCs, and CFs—and examined underlying mechanisms of the observed effects. CFs have a more substantial influence on APD than hMSCs and hCICs, resulting from their larger sink effect and total ionic outward current (Figure 5). The culprit is likely the CF delayed rectifier current (G_*kv*_), which showed the largest magnitude negative correlation with APD (Figure 6). In comparison, hCICs and hMSCs caused a lesser decrease in APD, and increased calcium transient amplitude by positively impacting the net flux through LTCC and NCX (Figure 6). Mechanistically, hCIC and hMSC coupling lowered membrane voltage during the plateau phase (Figure 4), which was closer to the optimal peak I_*LCa*_ that has previously been described (O’Hara et al., 2011).

In addition, we compared individual non-excitable cell treatment to CardioChimeras and combination hMSC-hCIC therapies given the recent advancements in these hybrid cell-based approaches (Williams et al., 2013; Quijada et al., 2015; Firouzi et al., 2020; Bolli et al., 2021). As shown in Figure 4, our simulation predicts HC-only treatment of healthy and diseased hCMs with CardioChimeras is less beneficial to calcium cycling than treating with each cell type alone, presumably due to the larger total ionic current density caused by combining both hMSC and hCIC currents at a capacitance similar to an individual cell. Also, the combined hMSC-hCIC effect was intermediate between treating with each cell type alone at the same dosage, likely due to a total ionic current density approximately averaged between the hMSC and hCIC currents. By contrast, Quijada et al. (2015) found ejection fraction was significantly higher at 6 weeks post-myocardial infarction injury for mice treating with two distinct CardioChimera cell lines, as well as combined MSCs and CICs, but not for mice treated with hMSCs or hCICs alone at the same dosage, in comparison to the placebo group. Given this discrepancy, we therefore hypothesized that HC alone simply cannot explain the beneficial effects of treating HF-hCMs or FHF-hCMs with hCICs and hMSCs in combination or as CardioChimeras, and instead another predominant cell-based mechanism in the literature—namely paracrine signaling—must be more contributory.

To test this hypothesis, we used a population-based method to assess which combinations of hMSC and/or hCIC treatments, and which underlying mechanisms are most potent in restoring electrical and calcium cycling behavior of HF-hCMs and FHF-hCMs back toward healthy hCMs. Indeed, as shown in our *in silico* permutation experiments, paracrine signaling rather than HC appears to be a key mechanism underlying the pro-contractile benefits of cell-based heart therapy; this appears to reflect anti-fibrotic paracrine effects, since FHF-hCMs but not HF-hCMs were correctable to near-healthy conditions. The *in silico* treatment optimization not only maximized the restorative benefits, but also appeared to minimize pro-arrhythmic risks of the cell therapies (Figures 9, 10). Notably, the accepted model populations yielded a smaller range of potentially detrimental RMPs and UVs, and simulations of PS-only treatment helped restore RMPs and UVs of failing fibrotic cardiomyocytes to near-healthy values. Such theoretical advantage of hMSC paracrine signaling complements the findings by Quijada et al. (2015), which showed that the beneficial cardiac contractile effects of CardioChimeras and combined MSC and CIC treatment were not associated with cell engraftment into the myocardium. Altogether, these findings are consistent with paracrine signaling playing a predominant role in cardiac cell therapy, and highlight the need for further research into secretome-based therapeutics.

Our findings are consistent with an even wider range of *in vitro* (Mayourian et al., 2017), *in vivo* (Luo et al., 2017; Tang et al., 2017), and clinical data (Mathiasen et al., 2019; Bolli and Kahlon, 2020). For example, our group recently showed that hCIC supplementation improves contractile performance of human engineered cardiac tissues, in agreement with our simulation from Figure 4A (Murphy et al., 2019). Further, our group recently showed that the hMSC secretome, rather than HC, improves contractile performance of human engineered cardiac tissues (Mayourian et al., 2017), and that this occurs primarily through exosomal microRNA-21-5p, which is known to be anti-fibrotic and anti-apoptotic (Mayourian et al., 2018b). In fact, the importance of exosomal microRNA-21-5p was further highlighted by Qiao et al. (2019) when they showed cardiac stromal cell-derived exosomal microRNA-21-5p is reduced in heart failure patients, thus impairing regenerative potential via the PTEN/Akt pathway, the same pathway we found to be modulated by hMSC exosomal microRNA-21-5p. More recently, the Cheng group fabricated synthetic hMSC microparticles containing respective paracrine factors that reduced left ventricular remodeling in a mouse model of acute myocardial infarction (Luo et al., 2017; Tang et al., 2017). The same group showed a cardiac patch composed of a decellularized porcine myocardial extracellular matrix scaffold and encapsulated secreted factors from isolated human cardiac stromal cells reduced scarring, boosted cardiac function, and promoted angiogenesis in a rat model of acute myocardial infarction (Huang et al., 2020). Finally, in clinical trials, the anti-fibrotic effects of hMSCs and hCICs have been well established (Hare et al., 2012; Heldman et al., 2014; Karantalis et al., 2014; Tang et al., 2016; Mathiasen et al., 2019; Bolli and Kahlon, 2020), and interestingly, a recent clinical trial noted that hMSC effects are dose-dependent (Mathiasen et al., 2019), in agreement with our findings in Figure 8E where calcium transient amplitude is dependent on the paracrine signaling dosage.

In summary, this work complements previous studies and suggests future research should focus on strategies to harness and deliver cardioactive elements of the hMSC and hCIC secretome. As discussed above, several groups have started down this avenue (Luo et al., 2017; Tang et al., 2017), and if proven effective, such strategies to optimize the delivery of key paracrine factors may offer advantages for off-the-shelf therapeutics that capture the benefits of stem cell therapy while circumventing the potential risks and challenges associated with delivering live biologics to the heart.

### Limitations and Future Directions

One limitation of this study was the paucity of experimental data available on which to build a CIC electrophysiology model. While studies of canine and rat CICs have shown distinct ion channel profiles (Han et al., 2010; Khalafalla and Sussman, 2018; Vigneault et al., 2018), given our interest in translational human work, we focused specifically on human CICs in isolation. Therefore, the study by Zhang et al. (2014) served as the sole basis for our model development. Further model validation will be valuable as additional experimental data become available. For example, the Li laboratory subsequently identified transient receptor potential vanilloid channels inside of hCIC capable of conducting a calcium influx when activated (Che et al., 2016). To date, we have only incorporated a non-specific leakage current that encompasses the actions of these receptors and numerous others yet to be defined. Additional experimental data on the intracellular calcium dynamics in these cell populations is required for the development of more nuanced models of non-excitable cells in the future. Likewise, our CardioChimera model was limited by the lack of available empirical ion channel data, leading us to assume the hybrid cell could be modeled by a simple additive combination of hMSC and hCIC functional activity, which requires experimental validation.

Second, in this study we assumed hCIC PS does not alter cardiomyocyte electrophysiology or calcium cycling. This is consistent with observations by Smit et al. (2017) that hCIC conditioned media had no significant effect on rat cardiomyocyte action potential morphology, and others found the exosomal component of the hCIC secretome to have limited uptake by cardiomyocytes with no significant effect on cardiomyocyte calcium cycling (Gray et al., 2015). This assumption guided us to exclude hCIC PS in our model permutation experiment. However, while hCIC PS may not alter cardiomyocyte electrophysiology or calcium cycling directly, it may influence hMSC survival, paracrine potency, or engraftment (Williams et al., 2013; Quijada et al., 2015), thereby impacting HC, a mechanism our current model does not yet address. Furthermore, hCIC PS is known to have anti-fibrotic effects (Quijada et al., 2015), which could be incorporated into future computational models as we have described (Mayourian et al., 2017), when dose-dependent experimental data become available. We further acknowledge that our current hMSC paracrine model is empirically based on *in vivo* data of MSC delivered at different dosages and the resultant anti-fibrotic effects, but it does not reflect the underlying mechanism leading to these anti-fibrotic effects; recent advancements have shown that in addition to the anti-fibrotic nature of hCIC and/or hMSC paracrine factors alone (Luo et al., 2017; Tang et al., 2017), the act of cell delivery itself leads to an acute inflammatory-based wound-healing response that rejuvenates the infarcted area of the heart (Vagnozzi et al., 2020). As this fascinating field continues to develop, our model can be improved accordingly to not only incorporate hCIC paracrine effects but also fibroblast and immune cell signaling effects on cardiomyocytes.

Third, we only provide single-cell metrics of arrhythmogenicity, as tissue-level analysis was beyond the scope of the current study. Importantly, we showed that hCIC and/or hMSC coupling resulted in elongation of APD triangulation, resting depolarization, and decreased UV, which may indicate pro-arrhythmic potential. Arrhythmogenic risk of hMSCs has been addressed previously by our group using tissue-level analysis (Mayourian et al., 2016, 2017), and a similar approach could be used with the new hCIC model when the relevant experimental data become available. Such efforts could use more recent healthy cardiomyocyte models that have been revised to account for limitations in the LTCC, and may be better suited to assess arrhythmogenic risk (Tomek et al., 2019). Addressing this limitation may be clinically relevant, as the recent results of Bolli et al. (2021) showed a higher incidence of ventricular arrhythmias in all cell therapy groups, with the highest rate of occurrence with isolated hCIC treatment (albeit these findings were not statistically significant). Future computational work should examine the arrhythmogenicity of combined hCIC and hMSC treatment, and assess whether delivery of paracrine factors could be a strategy to harness the benefits while minimizing potential risks of live cell-based cardiotherapies, as suggested by this study.

Fourth, we note that the stringent acceptance criteria for our permutation experiments could lead to false negatives; for example, it is feasible that the restoration of calcium cycling alone, as shown in Figures 7A,B, may be sufficient to provide meaningful benefit despite not meeting our predefined acceptance criteria (Equation 6) for both calcium cycling and action potential metrics.

Finally, we acknowledge that although mathematical models offer the capability to explicitly control variables that can be challenging to manipulate experimentally, they are also subject to the limitations of their underlying assumptions, and ultimately the most valuable insights will come from a synergistic combination of computational modeling and experimental validation.

## Data Availability Statement

The raw data supporting the conclusions of this article will be made available by the authors, without undue reservation.

## Author Contributions

KP, IT, RH, KC, and JM: responsible for substantial contributions to the study conception and design. KP and JM: computational modeling, data acquisition, data analysis, and drafting of the manuscript. KC, IT, RH, and JM: critical revision of the manuscript. All authors contributed to the article and approved the submitted version.

## Conflict of Interest

KC discloses his role as scientific co-founder and Chief Scientific Officer of NovoHeart Ltd. NovoHeart did not play any role in the design or conduct of this study. The remaining authors declare that the research was conducted in the absence of any commercial or financial relationships that could be construed as a potential conflict of interest.

## Publisher’s Note

All claims expressed in this article are solely those of the authors and do not necessarily represent those of their affiliated organizations, or those of the publisher, the editors and the reviewers. Any product that may be evaluated in this article, or claim that may be made by its manufacturer, is not guaranteed or endorsed by the publisher.

## Abbreviations

APD_x_: Action potential duration at x% repolarization
APD_tri_: Action potential duration triangulation
[Ca^2+^]_i,amp_: Calcium transient amplitude
[Ca^2+^]_*i*,max_: Peak systolic calcium
[Ca^2+^]_*i*,min_: Diastolic calcium
C_*cell*_: Membrane capacitance
CC: hCIC-hMSC CardioChimera
CF: Cardiac fibroblast
I_Dr_: hMSC delayed rectifier channel
G_x_: Conductance of channel x
HC: Heterocellular coupling
hCIC: Human c-kit ^+^ cardiac interstitial cell
hCM: Human cardiomyocyte
HF-hCM: Heart failure human cardiomyocyte in non-fibrotic myocardium
FHF-hCM: Heart failure human cardiomyocyte in fibrotic myocardium
hMSC: Human mesenchymal stem cell
I_ion_: Sum of active ionic currents
I_KCa_: Large Conductance Ca^2+^-activated K^+^ channel
I_Kir_: Inward rectifying K^+^ channel
I_Na_: sodium channel
LTCC: L-type calcium channel
NCX: Sodium-calcium exchanger
PS: Paracrine signaling
Q_x_: Net charge for ionic current x
RMP: Resting membrane potential
SERCA: Sarcoendoplasmic reticulum calcium-ATPase
I_to_: Transient outward K^+^ channel
UV: Upstroke velocity
V_peak_: Peak action potential voltage
τ_ca_: Calcium relaxation time constant.
