# Supplementary material for: In silico Cell Therapy Model Restores Failing Human Myocyte Electrophysiology and Calcium Cycling in Fibrotic Myocardium
Source: Front Physiol. 2022 Jan 3;12:755881. doi: 10.3389/fphys.2021.755881 (PMC8762340; doi:10.3389/fphys.2021.755881)
Supplement: Supplementary file 1 [file Data_Sheet_1.pdf]

SUPPLEMENTARY MATERIAL ONLINE

Supplementary Tables:

Supplemental Table S1: hCIC Model Equations

**Large-Conductance Ca^{2+} Activated K^+ Channel**
$I_{KCa} = G_{KCa}x(V-E_K)$ where G_{KCa} =6.62nS and E_K=-89.1mV <br>$\frac{dx}{dt} = \frac{x_\infty - x}{\tau_x}$ <br>$x_\infty = \frac{1}{1+e^{\frac{V-V_{1/2}}{K}}}$ where $V_{1/2}$=-68.24mV and K=-17.06mV <br>$\tau_x = Ae^{-\left(\frac{V-V_{1/2}}{K}\right)^2} + B$ where A=9.98ms, B=4.26ms, $V_{1/2}$=93.20mV and K=28.90mV
**Inward Rectifier K^+ Channel**
$I_{Kir} = G_{Kir}\sqrt{\frac{K_o}{5.4}}\,x_{Kir\infty}(V-E_K-V_1)$ where G_{Kir}=1.2nS, K_o=5, E_K=-68mV, V_1=77.2mV <br>$x_{Kir\infty} = \frac{\alpha_{Kir}}{\alpha_{Kir}+\beta_{Kir}}$ <br>$\alpha_{Kir} = \frac{A}{1+e^{B(V-E_K+V_1)}}$ where A=4.84, B=-0.058, E_K=-68mV and V_1 = -13.1mV <br>$\beta_{Kir} = \frac{Ae^{B(V-E_K-V_1)}+e^{C(V-E_K-V_2)}}{1+e^{D(V-E_K-V_3)}}$ where A=0.584, B=0.055, C=-0.048, D= -0.13, E_K=-68mV, V_1=-35.0mV, V_2=3mV, and V_3=-7.6mV

**Transient Outward K^+ Channel**
$I_{to}=G_{to,1}(V-E_K)+G_{to,2}r_{sus}(V-E_K)$ where $G_{to,1}$=12.02nS, $G_{to,2}$=0.62nS and E_K=-89.1mV <br>$\frac{dr}{dt}=\frac{r_\infty - r}{\tau_r}$ <br>$\frac{ds}{dt}=\frac{s_\infty - s}{\tau_s}$ <br>$r_\infty=\frac{1}{1+e^{\frac{V-V_{1/2}}{K}}}$ where $V_{1/2}$=108.9mV and K=-36.5mV <br>$s_\infty=\frac{1}{1+e^{\frac{V-V_{1/2}}{K}}}$ where $V_{1/2}$=-14.5mV and K=7.9mV <br>$r_{sus,\infty}=\frac{1}{1+e^{\frac{V-V_{1/2}}{K}}}$ where $V_{1/2}$=16.6mV and K=-10.2mV <br>$\tau_r=Ae^{-\left(\frac{V}{V_1}\right)^2}+B$ where A=24.5ms, B=2.48ms, and V_1=26.6mV <br>$\tau_s=Ae^{-\left(\frac{V-V_1}{V_2}\right)^2}+B$ where A=57.1ms, B=19.5ms, V_1=27.7mV, and V_2=11.3mV <br>$\tau_{r,sus}$= 5ms
**Na^+ Channel**
$I_{Na}=G_{Na}mh\,(V-E_{Na})$ where G_{Na}=0.105nS and E_{Na} = 80.0mV <br>$\frac{dm}{dt}=\frac{m_\infty - m}{\tau_m}$ <br>$\frac{dh}{dt}=\frac{h_\infty - h}{\tau_h}$

$$m_\infty = \left(\frac{1}{1+e^{\frac{V-V_{1/2}}{K}}}\right)^6$$

where $V_{1/2}$=-37.1mV and K= -7.85mV

$$h_\infty = \frac{1}{1+e^{\frac{V-V_{1/2}}{K}}}$$

where $V_{1/2}$=-44.5mV and K= 12.8mV

$$\tau_m = Ae^{-\left(\frac{V-V_{1/2}}{K}\right)^2} + B$$

where A=6.83ms, B=0.60ms, $V_{1/2}$=-27.88mV and K= 21.86mV

$$\tau_h = Ae^{-\left(\frac{V-V_{1/2}}{K}\right)^2} + B$$

where A=11.1ms, B=2.4ms, $V_{1/2}$=-15.3mV and K= 30.7mV

Leak Current

$$I_{leak} = A(V - E_{Ca})$$

where A=0.005nS and E_{Ca}=134mV

Total Current (Weighted by Prevalence)

$$I_{tot} = 0.61I_{Na} + 0.84I_{Kir} + 0.86I_{KCa} + 0.47I_{to} + I_{leak}$$

Supplemental Table S2: hMSC Paracrine Effect Parameters

Parameter	Value
ΔI_{CaL}	0.59
ΔJ_{up}	0.97
$EC_{50,CaL}$	$Log_{10}(35.3)$ n hMSCs
$EC_{50,Jup}$	$Log_{10}(6.6)$ n hMSCs
k_{CaL}	1.33/n hMSCs
k_{Jup}	4.19/n hMSCs

Supplemental Table S3: Model Optimization Search Space and Justification

Given the wide range of parameters that could be used for model optimization, exploratory tests were performed to identify a range of input parameter values that would place reasonable bounds on the optimization problem.

Search Space	Justification
Main Figures 6 and 7A-D:<br>Ratio of non-excitable cells (n_{hCIC} and n_{hMSC}) to cardiomyocyte included integer values between 0:1 and 4:1 (ie, 0:1, 1:4, 1:3, 1:2, 1:1, 2:1, 3:1, 4:1). n_{hCIC} and n_{hMSC} were randomly varied independently.	***Main Figures 6 and 7:***<br>In exploratory tests, coupling more than 4 non-excitable cells per myocyte would begin to reduce calcium transients from untreated conditions, deviating further from the healthy target condition. Therefore, to reduce computational expense, a maximum total of 4 non-excitable cells was selected.
Main Figures 6 and 7:<br>Gap junctional conductance of 10nS or less as a continuous variable<br>($0.0 \leq G_{junction} \leq 10.0$nS)	***Main Figure 6 and 7:***<br>As shown in Figure 3, a gap junctional conductance of 10nS had very similar results to the fusion condition with infinite conductance. Therefore, a maximum gap junctional conductance of 10nS was selected to reduce computational expense.
Main Figure 6:<br>Number of hMSC paracrine effects were set equal to number of hMSC heterocellular coupling effects ($n_{hMSC,PS} = n_{hMSC,HC}$). $n_{hMSC,PS}$ had the same search space as n_{hMSC} above (ie, 0:1, 1:4, 1:3, 1:2, 1:1, 2:1, 3:1, 4:1).<br><br>***Main Figure 7A-D:***<br>Number of hMSC paracrine effects ($n_{hMSC,PS}$) randomly varied independent of the number of hMSC heterocellular coupling effects ($n_{hMSC,HC}$). $n_{hMSC,PS}$ had the same search space as n_{hMSC} above (ie, 0:1, 1:4, 1:3, 1:2, 1:1, 2:1, 3:1, 4:1).	***Main Figure 6:***<br>In exploratory tests, including paracrine signaling effects of more than 4 hMSC per myocyte would overshoot normal calcium transient amplitude. Therefore, a maximum PS effect equivalent to 4 hMSCs was selected to reduce computational expense. In addition, the experimental design for this figure was to simulate effects of delivering non-excitable cells, which would lead to equivalent number of hMSCs exerting both HC and PS effects<br><br>***Main Figure 7A-D:***<br>In exploratory tests, including paracrine signaling effects of more than 4 hMSC per myocyte would overshoot normal calcium transient amplitude. Therefore, a maximum PS effect equivalent to 4 hMSCs was selected to reduce computational expense. In this figure, the experiment was designed to identify the relative effects of HC versus PS; therefore, $n_{hMSC,PS}$ and $n_{hMSC,HC}$ were varied independently.

Main Figure 7E: Number of hMSC paracrine effects ($n_{hMSC,PS}$) was a continuous variable between 0 and 4.	***Main Figure 7E:*** In exploratory tests, including paracrine signaling effects of more than 4 hMSC per myocyte would overshoot normal calcium transient amplitude. Therefore, a maximum PS effect equivalent to 4 hMSCs was selected to reduce computational expense.

Supplementary Figures:

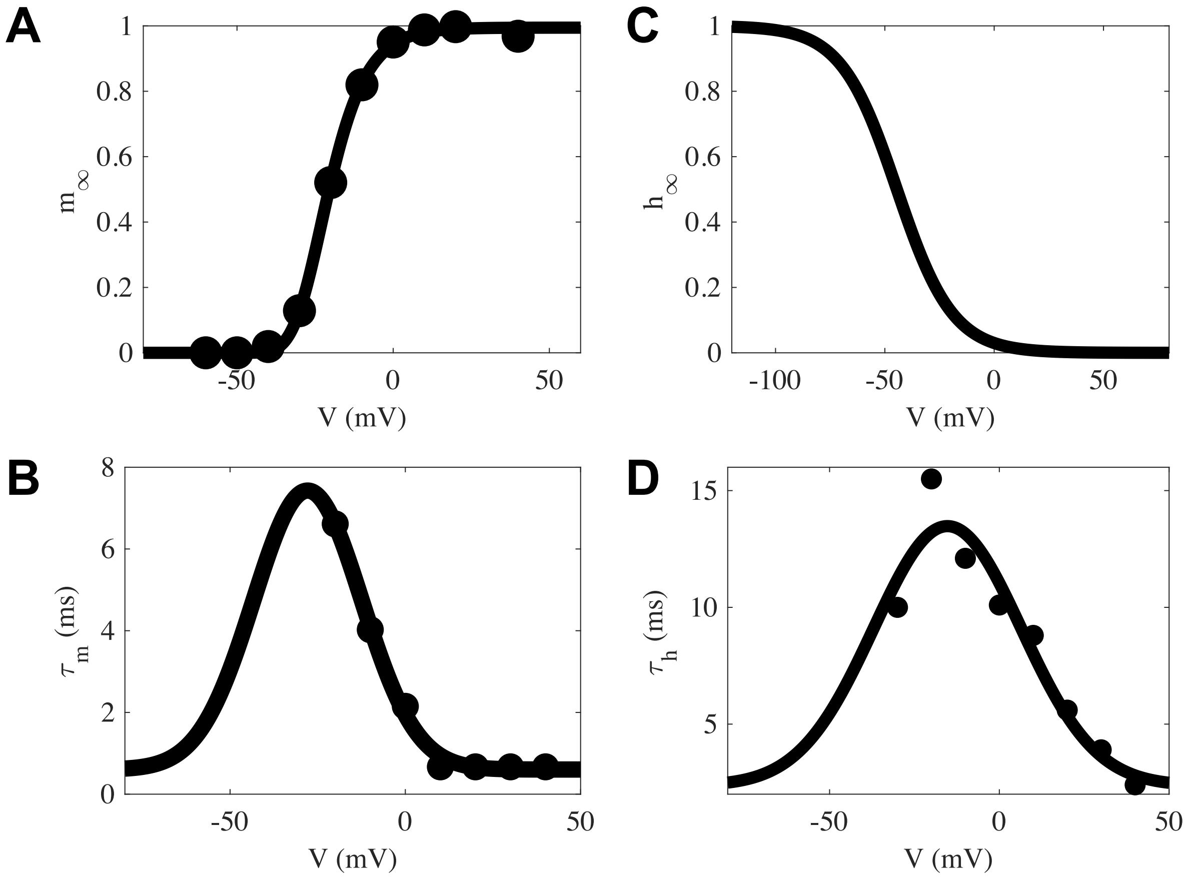

Supplemental Figure S1: I_{Na} Steady-State Functions and Time Constant Curves: Steady-state (A) activation and (C) inactivation curves for I_{Na} together with values derived from digitizing I-V data (Zhang et al., 2014). Note that the inactivation curve equation was already provided within Zhang et al., explaining the lack of experimental data points. Time constant (B) activation and (D) inactivation curves for I_{Na} together with values derived from digitizing voltage-clamp data (Zhang et al., 2014).

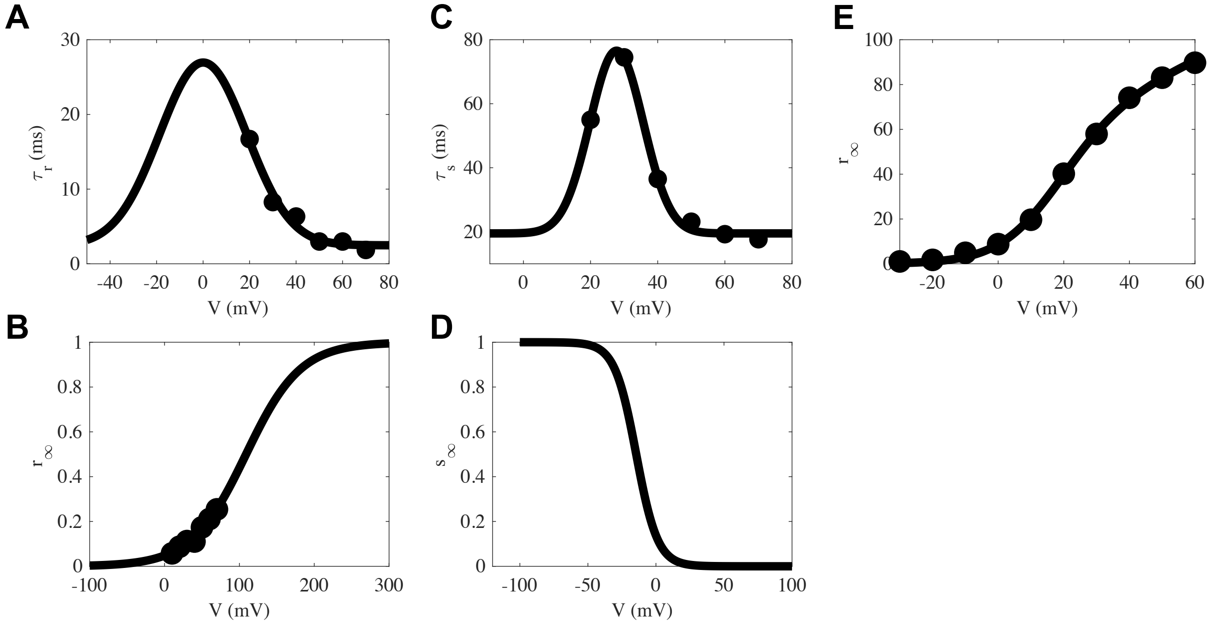

<u>Supplemental Figure S2:</u> I_{to} Steady-State Functions and Time Constant Curves: Steady-state (B) activation and (D) inactivation curves for I_{to} together with values derived from digitized experimental data (Zhang et al., 2014). Note that the inactivation curve equation was already provided within Zhang et al., explaining the lack of experimental data points. Time constant (A) activation and (C) inactivation curves for I_{to} together with values derived from digitizing voltage-clamp data (Zhang et al., 2014). (E) Steady-state activation curve for I_{to} sustained currents together with values derived from digitizing voltage-clamp data (Zhang et al., 2014).

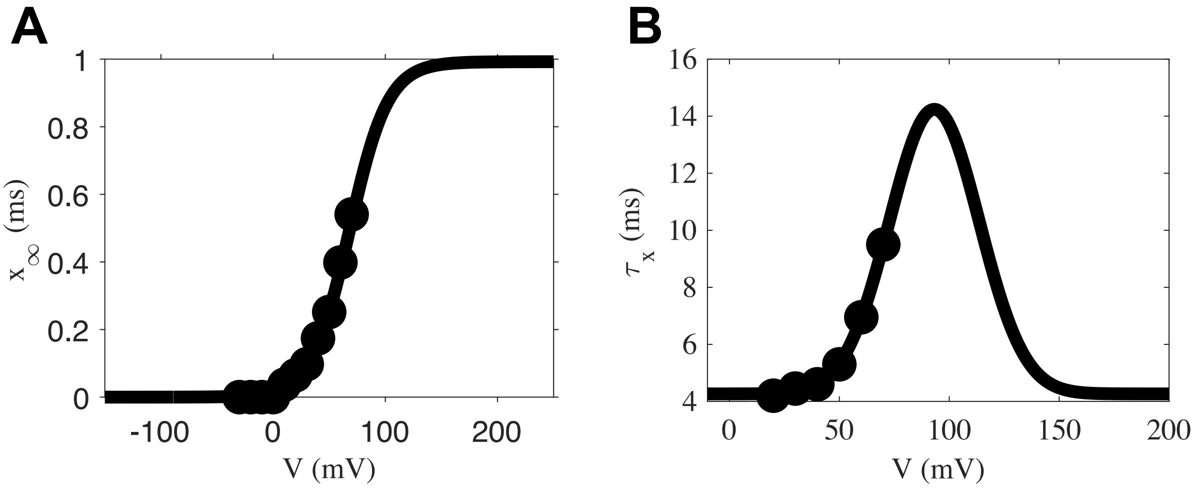

Supplemental Figure S3: I_{KCa} Steady-State Activation and Time Constant Curves: (A) Steady-state activation curve for I_{KCa} together with values derived from digitized mean I-V experimental data (Zhang et al., 2014). (B) Time constant curve for I_{KCa} together with values derived from digitized experimental voltage clamp data (Zhang et al., 2014).

Supplementary References:

1. Zhang, Y.Y., Li, G., Che, H., Sun, H.Y., Li, X., Au, W.K., Xiao, G.S., Wang, Y., and Li, G.R. (2014). Characterization of functional ion channels in human cardiac c-kit+ progenitor cells. Basic Res Cardiol 109, 407.
